# Why do patients with isolated PCL rupture experience no subjective knee joint instability during walking? An *in vivo* biomechanical study

**DOI:** 10.3389/fbioe.2024.1495266

**Published:** 2025-01-07

**Authors:** Mingfeng Lu, Wei Chen, Jinpeng Lin, Wenhan Huang, Junqing Gao, Lilian Zhao, Shilin Li, Lilei He, Yu Zhang

**Affiliations:** ^1^ The Eighth Clinical Medical College, Guangzhou University of Chinese Medicine, Foshan City, China; ^2^ Department of Sports Medicine, Foshan Hospital of Traditional Chinese Medicine, Foshan, China; ^3^ Department of Rehabilitation Therapy Teaching and Research, Gannan Health Vocational College, Gan Zhou, China; ^4^ School of Materials Science and Engineering (National Engineering Research Center for Tissue Restoration and Reconstruction), South China University of Technology, Guangzhou, China; ^5^ Department of Orthopaedics, Guangdong Provincial People’s Hospital (Guangdong Academy of Medical Sciences), Southern Medical Universit, Guangzhou, China; ^6^ Department of Reparative and Reconstructive Surgery, Foshan Hospital of Traditional Chinese Medic, Foshan, China

**Keywords:** posterior cruciate ligament, joint instability, kinematic, gait, biomechanics

## Abstract

**Objective:**

The aim of this study is to assess the kinematic changes in the knee joint during walking in patients with isolated PCL-deficiency (PCLD) to determine the presence of walking-related joint instability (mechanical instability—abnormal displacement form structural damage). Additionally, the study seeks to provide biomechanical insights into the observed differences between subjective and objective assessments.

**Methods:**

35 healthy volunteers and 27 patients with isolated PCLD (both involved and uninvolved sides) were included in the study. All participants walked on a treadmill at a self-selected comfortable speed. An optical 3D motion capture system was employed to collect six degrees of freedom kinematic data of the knee joint during walking. Statistical Parametric Mapping (SPM) was employed, using independent and paired t-tests to evaluate differences between the healthy control group and the PCLD group, as well as between the involved and uninvolved sides, respectively.

**Results:**

Compared with the healthy control group, posterior tibial displacement (the main indicator for anterior-posterior instability) of the involved limb was significantly decreased during 79%–94%. additionally, knee flexion angles of the involved limb were significantly increased compared with healthy control group during 0%–5% and 95%–99% of the gait cycle and significantly decreased during 66%–87%; In the uninvolved side, adaptive gait changes were observed, with knee flexion angles significantly reduced during 20%–50% and 64%–89% of the gait cycle and posterior tibial displacement significantly reduced during 60%–94% compared with the healthy control group; Compared to the uninvolved limb, the involved limb showed increased internal rotation during 62%–71% of the gait cycle and increased knee flexion during 8%–53%, with no significant differences in other dimensions.

**Conclusion:**

From a biomechanical perspective, patients with PCL rupture exhibit no joint instability during walking. Compared to the healthy control group, the involved leg shows a significant reduction in posterior tibial displacement and a diminished range of knee flexion. Clinical evaluations of PCLD should incorporate dynamic functional assessments, thereby providing a more comprehensive basis for treatment decisions.

## 1 Introduction

The Posterior Cruciate Ligament (PCL) plays a critical role in maintaining knee joint stability by restricting the posterior displacement of the tibia relative to the femur and contributing significantly to the rotational stability of the knee (Castle et al., 1992; Arthur et al., 2020). PCL injuries are common in both vehicular accidents and sports, accounting for 3%–44% of all knee joint injuries (Harner and Höher, 1998; Schulz et al., 2003). The changes in joint stability and mechanical load will be caused by PCL injury (Chandrasekaran et al., 2012).

However, clinicians often encounter discrepancies between objective examination results and patients’ subjective experiences. Assessment methods of PCL injuries include special tests such as the Posterior Drawer Test, Posterior Sag Test, Hopkin’s Test, KT2000, as well as imaging techniques like MRI and arthroscopy (Kopkow et al., 2013). In patients with PCL-deficiency (PCLD), these tests typically yield positive results. Nevertheless, it is perplexing that many patients who show positive test results do not report experiencing knee instability during routine walking. Keller et al. (1993) conducted a questionnaire survey involving 40 patients with chronic, complete PCL injuries and found that 35 of them did not perceive knee instability during normal walking. It has been observed that the assessment of posterior knee instability based on these tests does not always align with patients’ subjective evaluations (Fowler and Messieh, 1987; Shelbourne et al., 1999; Shelbourne and Muthukaruppan, 2005). This discrepancy between subjective and objective assessment results has been a significant factor influencing patients’ choice of treatment (surgical reconstruction or conservative management). Therefore, exploring the kinematic patterns of the knee joint post-injury and determining whether objective knee instability exists in PCLD patients is crucial.

The discrepancies between subjective and objective evaluations may arise from the assessment methods used. Neither clinical special tests nor imaging techniques accurately replicate the active motion states of PCLD patients. *In vivo* biomechanical assessment of knee joint motion in PCLD patients may more precisely identify knee instability, as three-dimensional motion capture can objectively describe the entire motion process.

In previous *in vivo* biomechanical studies of PCLD, researchers have predominantly focused on joint angles and moments at the hip, knee, and ankle during various activities. For instance, Hooper et al. (2002) described the sagittal plane (flexion-extension) movement characteristics of PCLD patients during walking and stair climbing. Castle et al. (1992) investigated ground reaction forces and sagittal plane knee angles during squatting in PCLD patients. Fontboté et al. (2005) used three-dimensional motion capture to analyze knee joint angles and moments in the sagittal, frontal, and transverse planes during walking, standing, and vertical jumping in PCLD patients. Yu et al. (2019) applied a similar approach to study the three-dimensional motion characteristics of the entire lower limb (hip, knee, and ankle) during the stance phase (0%–60% in gait cycle) (Gronley and Perry, 1984) of walking in PCLD patients. However, these studies mainly focused on the stance phase of walking and inadequately addressed the dimensions of knee joint displacement (displacement of the tibia relative to the femur). Besides restricting joint rotation, the PCL’s primary role is to limit significant posterior displacement of the tibia relative to the femur (Chahla et al., 2020). Analyzing tibial displacement in the anterior-posterior, proximal-distal, and medial-lateral directions is also crucial for understanding knee joint movement. Thus, examining the six degrees of freedom (6DOF) of knee movement during walking in PCLD patients may offer a more comprehensive assessment of joint motion.

We utilized an optical three-dimensional motion capture system (Chahla et al., 2020) to record the 6DOF of knee movement throughout the walking cycle. The rationale for selecting level walking is that it is a fundamental daily activity with a simple, controlled movement pattern, making it ideal for evaluating knee joint function. Level walking minimizes the influence of high-impact forces from activities like running or jumping, enabling a focused analysis of posterior cruciate ligament (PCL) injury on joint stability. Its biomechanical relevance has been highlighted in studies assessing knee kinematics and load distribution (Hubley-Kozey et al., 2021; Wu et al., 2020). The aim of this study is to identify whether PCLD patients exhibit knee instability during walking by comparing the involved and uninvolved legs of PCLD patients with a healthy control group. From a biomechanical perspective, this research investigates the reasons behind the discrepancies between clinical objective assessments and patient subjective experiences, providing a reference for clinical treatment decisions and improving rehabilitation training.

## 2 Methods

### 2.1 Subjects

**FIGURE 1:**
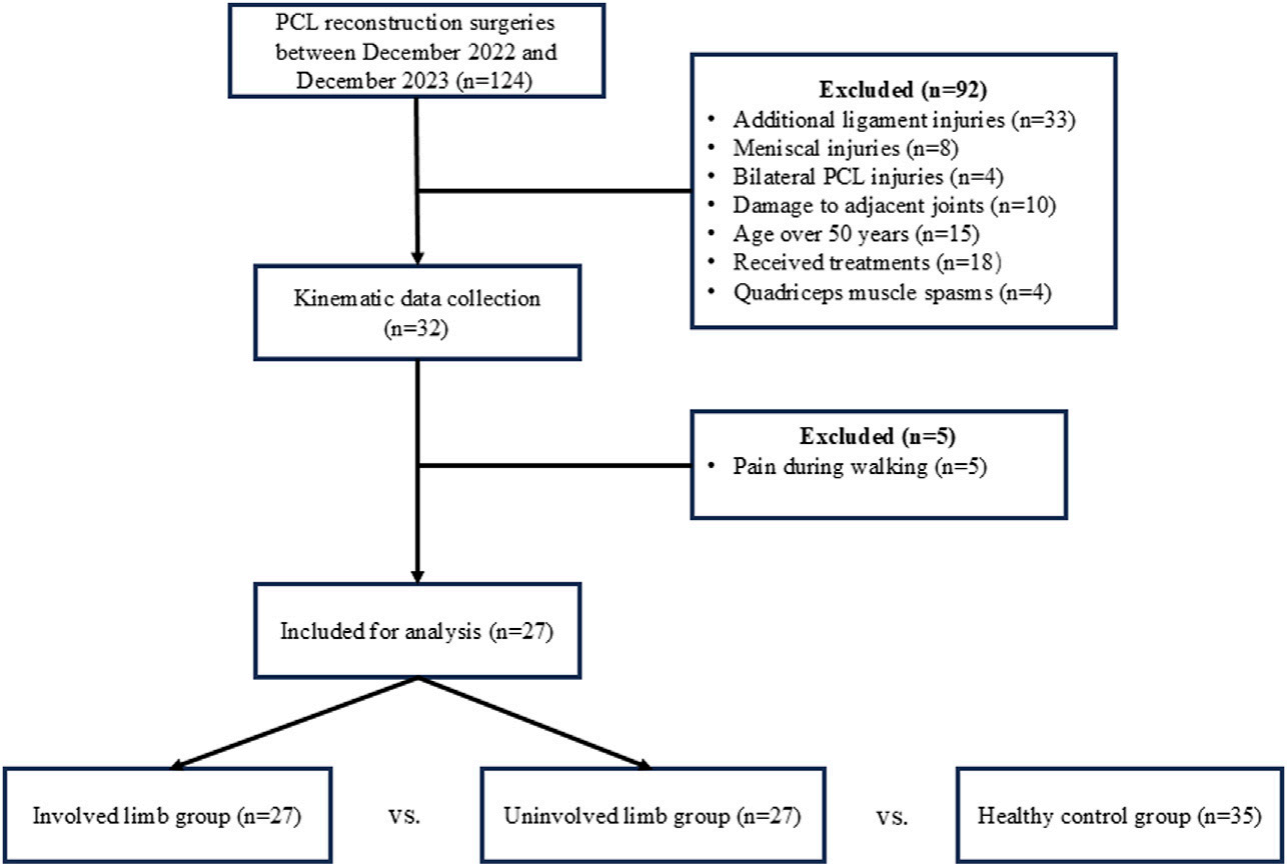
Patient flowchart throughout the study.

The experimental protocol was approved by the Ethics Committee of Foshan Hospital of Traditional Chinese Medicine. Informed consent was obtained from all individuals. 27 patients with unilateral complete isolated PCL ruptures, who visited to the hospital between December 2022 and December 2023, were included as the PCLD group (Figure 1). This group comprised 15 males and 12 females with a mean age of 24.3 years. Additionally, 35 healthy volunteers matched for gender, age, and BMI were recruited from the community as the control group. The 6DOF kinematics of one knee joint of the healthy control group was randomly selected for comparison with that of PCLD patients. There were no significant statistical differences in age and BMI between the PCLD and control groups (Table 1). PCLD patients were diagnosed by experienced clinicians using MRI, manual laxity testing, and range of motion assessments. Clinical examination findings were confirmed by arthroscopy within 0–6 days. Manual tests included the assessment of KT-1000 and the posterior drawer test.

**TABLE 1:** Clinical parameters for the PCLD group and control group.

Items	PCLD group	Control group	*p*-value
Gender (Male: Female)	15:12	17:18	0.75
Age (years)	24.3 ± 6.7	25.4 ± 5.7	0.83
Height (cm)	158.8 ± 13.3	160.5 ± 11.7	0.86
Weight (kg)	63.6 ± 10.8	61.8 ± 11.2	0.77
BMI (kg/m^2^)	21.2 ± 2.4	22.4 ± 1.6	0.59
Injury side			
Left	20		
Right	7		
Time from injury (months)	8.5 ± 4.9		
KT-1000 (%)			
0 to 2	0 (0)		
3 to 5	0 (0)		
6 to 10	9 (33.3)		
>10	18 (66.7)		
Posterior drawer (%)			
Negative	0(0)		
+	0(0)		
++	8 (29.6)		
+++	19 (70.4)		
IKDC subjective score	59.8 ± 9.8		
Tegner score	3.6 ± 1.2		
Lysholm knee score	53.7 ± 5.6		

Values are shown as mean ± SD (range); PCLD: posterior cruciate ligament deficiency; BMI, body mass index; IKDC, International Knee Documentation Committee.

Inclusion criteria for PCLD patients were: complete isolated PCL rupture for more than 3 months; no significant pain or instability during walking. Exclusion criteria were: presence of additional ligament or meniscal injuries in the knee; bilateral PCL injuries; serious damage to adjacent lower limb joints such as the hip, ankle, or the contralateral lower limb; age over 50 years (to avoid the impact of arthritis); received treatments such as physiotherapy; existence of quadriceps muscle spasms.

### 2.2 Clinical assessment

All patients underwent a clinical examination, including KT-1000 measurements (MEDmetric, San Diego, CA) (Rousseau et al., 2017) and the posterior drawer test (Wollschläger et al., 2021) conducted by the senior surgeon (MF.L.) at their initial hospital presentation. Scores on the Tegner activity scale (range: 0 [low activity level] to 10 [very high activity level]), the International Knee Documentation Committee (IKDC) (Yoon et al., 2023) subjective form (range: 0 [worst] to 100 [best]), and the Lysholm knee scoring scale (Yoon et al., 2023) (range: 0 [worst] to 100 [best]) were obtained.

### 2.3 Apparatus

**FIGURE 2:**
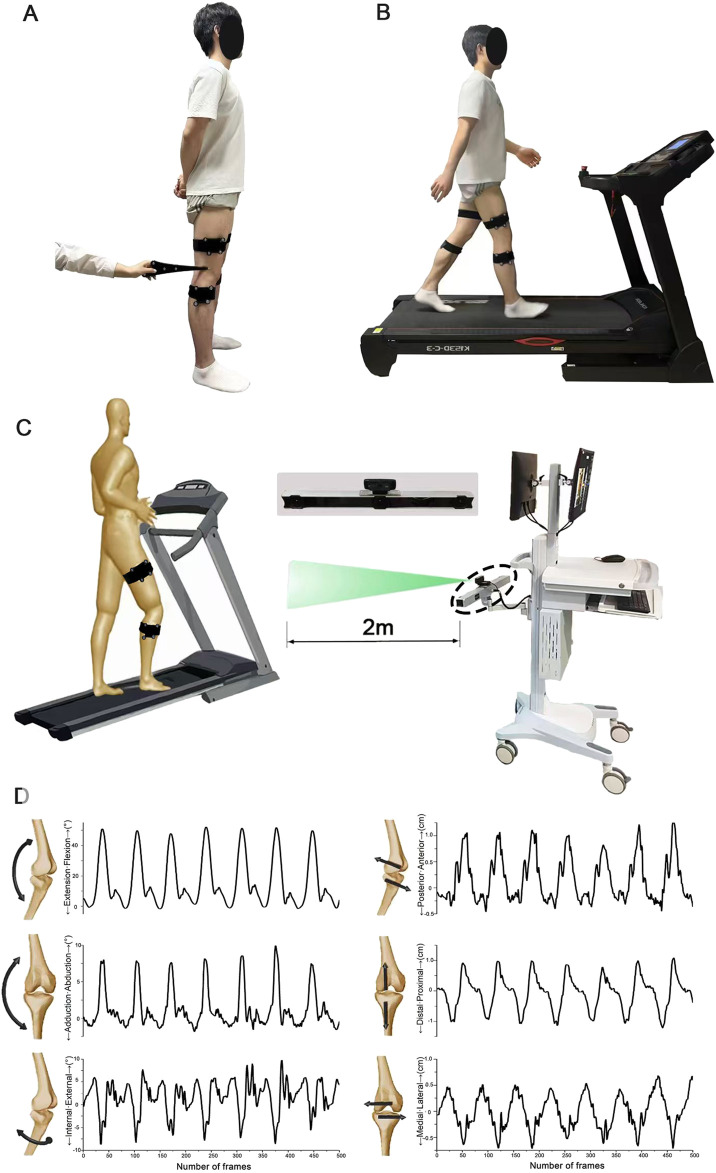
Schematic diagram illustrating the acquisition of six-degree-of-freedom gait parameters. **(A)** Calibration of bone markers facilitated by pointing to the hand-held probe for accurate positioning. **(B)** Walking conditions achieved using a bi-directional treadmill. **(C)** The scene depicting the collection of gait parameters, where marker clusters are identified and recorded by a high-speed camera to capture gait data. **(D)** Visualization of the six-degree-of-freedom knee kinematic data as the number of frames varies over time.

A three-dimensional motion capture system (Opti_knee, Innomotion Inc., Shanghai, China) was employed to record 6DOF kinematic changes in the knee during walking (Figure 2). This system includes a navigation-based stereo infrared tracking device (NDI Polaris Spectra; Northern Digital Inc.), high-speed optical cameras (Basler aca640–90uc; Basler AG), a rigid body placed on the femur, a rigid body placed on the tibia, and a handheld digital probe (Zhang et al., 2016). The motion capture system operates at a sampling frequency of 60 Hz. The accuracy level of the system is 0.3 mm root mean square (RMS),with repeatability of less than 1.3 degrees of rotation and 0.9 mm of translation (Elfring et al., 2010).

### 2.4 Testing procedures

Initially, all participants stood in a natural posture (feet shoulder-width apart, toes facing forward) with two rigid bodies attached to their thigh and shin using Velcro straps. A trained technician then used a digitizer to locate the anatomical landmarks on each participant’s lower limb, including the greater trochanter, medial and lateral femoral epicondyles, and the medial and lateral malleoli (Figure 2A shows the placement of our markers, with blue indicating the skeletal landmarks on the lower limb and green representing rigid bodies.). After marking all key points, the computer generated a 3D model of the lower limb bones based on the spatial positions of the calibration points. Secondly, participants stood on the treadmill, where the speed was gradually increased to allow them to acclimate to the environment and determine a comfortable walking speed (see Figure 2B). Finally, participants walked at their comfortable speed on the treadmill for 30 s per trial, with each trial repeated three times. In each trial, more than 10 complete gait cycles were ensured.

### 2.5 Statistical analysis

Demographic differences (age, gender, BMI) were assessed using independent samples t-tests and chi-square tests to determine statistical differences between the patient and control groups (SPSS version 24.0, IBM Corp., Armonk, NY, USA) (Chang et al., 2016). Kinematic data were normalized using MATLAB by averaging multiple gait cycles for each participant. Prior to analysis within the Statistical Parametric Mapping (SPM) library (Chang et al., 2016), all waveforms underwent normality testing. For comparisons between the uninvolved and involved limbs within the PCLD group, paired t-tests were conducted on normally distributed parameter waveforms using SPM. Independent t-tests were conducted to compare the involved leg of the PCLD group with control legs, as well as the uninvolved leg of the PCLD group with control legs, utilizing SPM for normally distributed parameter waveforms. Non-normally distributed parameters were analyzed using Statistical Non-Parametric Mapping (SnPM) with repeated measures Shapiro-Wilk tests. All SPM and SnPM analyses were conducted in MATLAB using the open-source spm1d (Version M.0.4.5; www.spm1d.org), with an alpha level of 0.05. SPM allows for the overall comparison of time-series data, identifying regions within waveforms where independent variables have significant effects and exceed critical thresholds. Significant regions were marked with *p*-values and the corresponding intervals.

## 3 Results

### 3.1 6DOF kinematic changes in the involved limb of PCLD patients during walking

**FIGURE 3:**
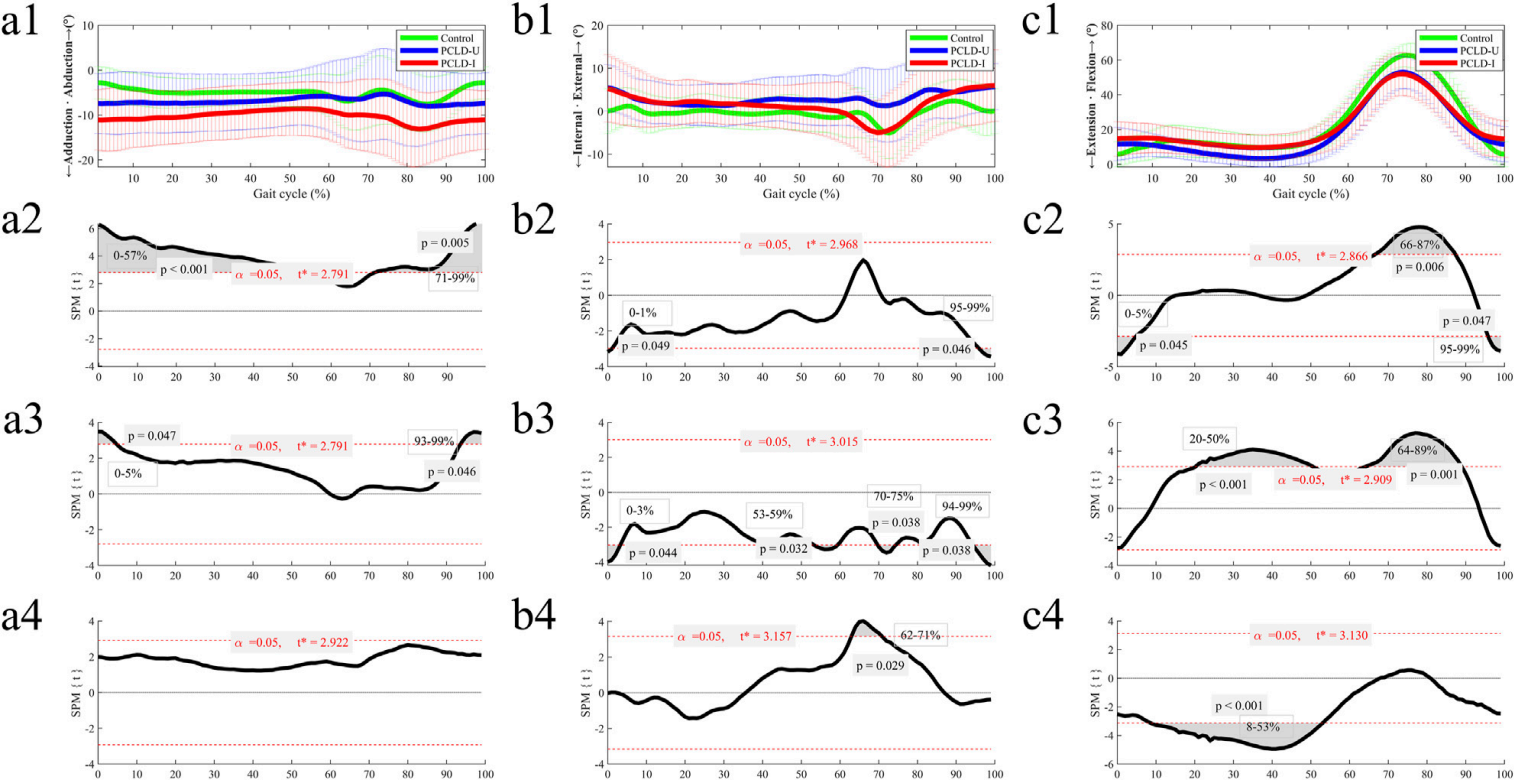
The ensemble average of knee angular kinematic comparisons of the Healthy control group, PCLD-U (uninvolved) group and PCLD-I (involved) group. Note: Charts a2, b2, c2 were the kinematic comparison between Healthy control group and PCLD-I (involved) group. Charts a3, b3, c3 were the kinematic comparison between Healthy control group and PCLD-U (uninvolved) group. Charts a4, b4, c4 were the kinematic comparison between PCLD-U (uninvolved) group and PCLD-I (involved) group.

**FIGURE 4:**
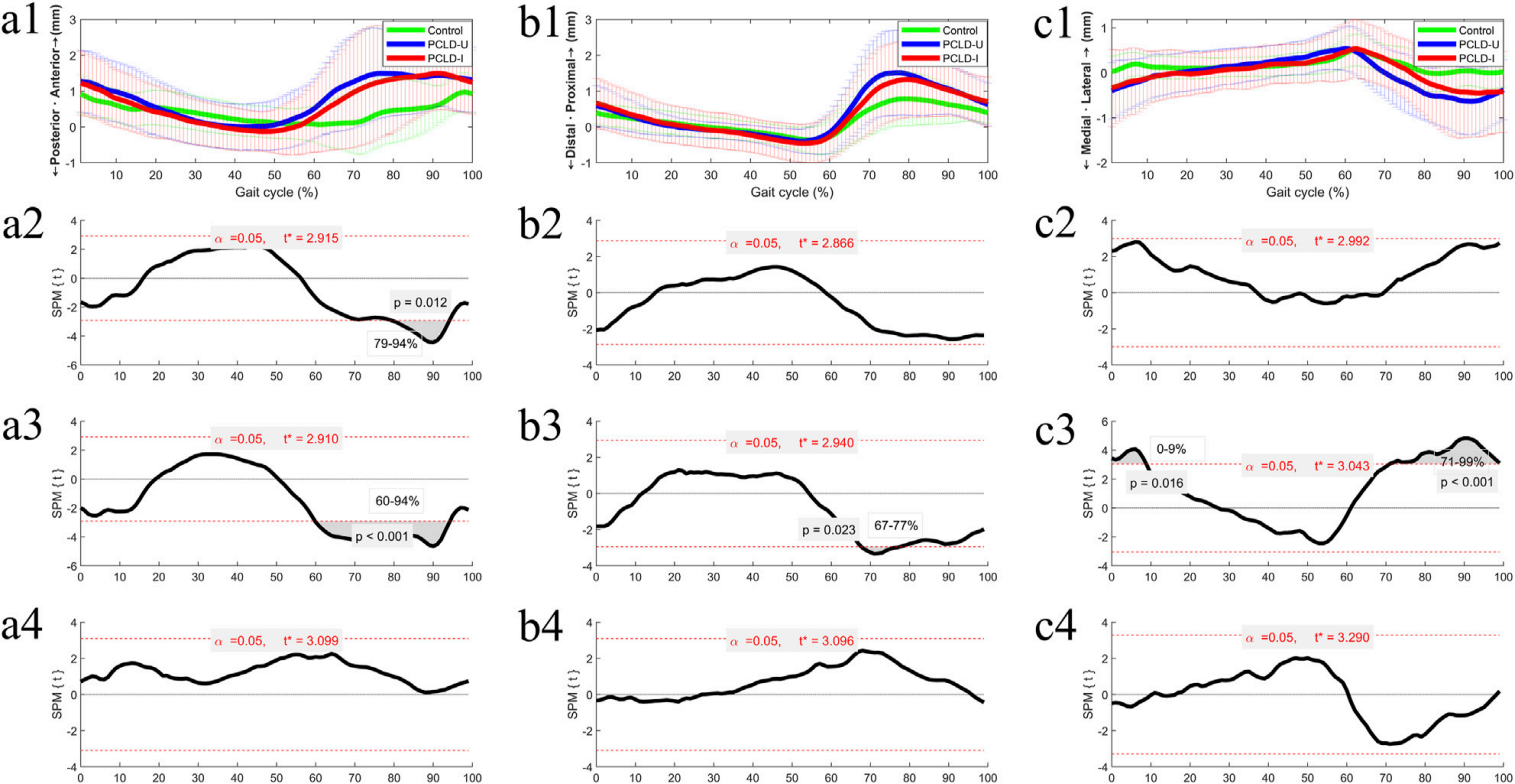
The ensemble average of knee translational kinematic comparisons of the Healthy control group, PCLD-U (uninvolved) group and PCLD-I (involved) group. Note: Charts a2, b2, c2 were the kinematic comparison between Healthy control group and PCLD-I (involved) group. Charts a3, b3, c3 were the kinematic comparison between Healthy control group and PCLD-U (uninvolved) group. Charts a4, b4, c4 were the kinematic comparison between PCLD-U (uninvolved) group and PCLD-I (involved) group.

The difference of 6DOF knee kinematics of healthy controls and PCLD patients were summarized in Figures 3, 4. Compared to the healthy control group, the involved limb of PCLD Patients exhibited significant increases in adduction during walking in the gait cycle phases of 0%–57% (*p* < 0.001) and 71%–99% (*p* = 0.005). These increases, while statistically significant, fall within the normal biomechanical range observed in healthy populations. Significant increases in external rotation were observed during the phases of 0%–1% (*p* = 0.049) and 95%–99% (*p* = 0.046) of gait cycle. Similarly, changes in transverse plane rotation likely reflect natural variability in joint kinematics. Regarding flexion-extension angles, the involved limb of PCLD Patients demonstrated significant increases in flexion during the phases of 0%–5% (*p* = 0.045) and 95%–99% (*p* = 0.047), while a decrease was noted during the 66%–87% phase (*p* = 0.0067). The observed changes in flexion-extension are consistent with compensatory adjustments during specific gait phases, maintaining functional joint performance. Posterior displacement of the tibia relative to the femur showed a significant decrease only during the 79%–94% of gait cycle, with no significant differences observed in other gait phases. This localized reduction in posterior displacement at late swing suggests that posterior stability is maintained overall during walking. Additionally, there were no significant differences in proximal-distal or medial-lateral relative displacements throughout the gait cycle. These findings suggest that although the joint kinematic characteristics of PCLD patients are different from those of healthy individuals, these changes are mainly manifested as functional compensation, and no evidence of mechanical or functional instability of the joint is observed. The knee joint maintains the necessary dynamic stability throughout the gait cycle.

### 3.2 6DOF kinematic changes in the uninvolved limb of PCLD patients during walking

Compared to the healthy control group, the uninvolved limb of PCLD Patients exhibited significant increases in adduction during the phases of 0%–5% (*p* = 0.047) and 93%–99% (*p* = 0.046) of gait cycle. These changes, though statistically significant, align with typical variations in gait mechanics. Significant increases in internal rotation angles were observed during the phases of 0%–3% (*p* = 0.044), 53%–59% (*p* = 0.032), 70%–75% (*p* = 0.038), and 94%–99% (*p* = 0.038). These rotational adjustments in the uninvolved limb suggest compensatory transverse plane alterations aimed at maintaining joint congruency and load distribution. Additionally, the flexion angles of the uninvolved limb were significantly reduced during the 20%–50% (*p* < 0.001) and 64%–89% (*p* = 0.001) phases. Reduced flexion during mid-stance and swing phases in the uninvolved limb may serve to counterbalance changes in the contralateral (involved) limb. In terms of tibial displacement relative to the femur, the uninvolved limb demonstrated a significant reduction in posterior displacement during the 60%–94% (*p* < 0.001) phase and a significant increase in proximal-distal relative displacement during the 67%–77% (*p* = 0.023) phase. Significant reductions in medial-lateral displacement were observed during the 0%–9% (*p* = 0.016) and 71%–99% (*p* < 0.001) phases. These findings highlight minor compensatory adjustments in spatial displacement. These results also indicate that the kinematics of the uninvolved limb in PCLD patients exhibit changes similar to those of the involved limb, including increased knee flexion during the stance phase of gait cycle, decreased knee flexion during the swing phase of gait cycle, and reduced posterior tibial displacement.

### 3.3 Comparison of 6DOF kinematic between the involved and uninvolved limbs in PCLD patients

Comparing the 6DOF kinematic between the involved and uninvolved limbs of PCLD patients during walking, it was observed that the involved limb had significantly reduced internal rotation angles during the 62%–71% (*p* = 0.029) phase and significantly increased knee flexion angles during the 8%–53% (*p* < 0.001) phase. No significant differences were found in the other four kinematic dimensions between the involved and uninvolved limbs.

## 4 Discussion

The objective of this study was to examine whether PCLD patients exhibit gait-related joint instability by assessing the kinematic changes in the knee joint during walking. Our most significant finding was that the posterior tibial displacement in the involved knee did not increase but instead significantly decreased during the swing phase of gait cycle. Additionally, the uninvolved limb of PCLD patients also exhibited corresponding adaptive changes. Our data indicate that mechanical knee joint instability does not occur during walking in PCLD patients. These results demonstrate that mechanical knee instability does not occur during walking in PCLD patients, aligning with their subjective reports of no perceived instability during this activity. This result helps explain the discrepancy between clinical assessments and subjective evaluations from a biomechanical perspective.

During the stance phase (0%–60%) of the gait cycle, the involved limb of PCLD patients did not exhibit significant differences in posterior tibial displacement compared to healthy controls, despite an increase in absolute displacement values. The results from vitro biomechanical studies on posterior tibial displacement relative to the femur have yielded varied findings. For example, Gollehon et al. (1987) observed that in 17 PCLD knee specimens, significant increases in posterior tibial displacement occurred only when knee flexion angles exceeded 60°. In contrast, Christopher (Harner et al., 2000) reported that in a comparison of 10 knee specimens with intact and PCLD conditions, significant differences in tibial posterior displacement were observed throughout knee flexion angles of 0°–120°. *In vivo* imaging studies, however, are more consistent with our findings. Ruediger (von Eisenhart-Rothe et al., 2012) using MRI scans of 12 PCLD patients and 20 healthy volunteers at knee flexion angles of 0°, 30°, and 90°, found significant differences in posterior tibial displacement only at 90° of knee flexion. Jonsson and Kärrholm (1999) reported that PCLD patients exhibited significantly increased posterior laxity (3.8–11.3 mm) when climbing stairs. Additionally, Castle et al. (1992) employed fluoroscopic techniques to assess tibial posterior displacement at various knee flexion angles and found significant differences only at knee flexion angles of 70°–90° (mean increase of 7.4 mm), with no significant differences at lower flexion angles. Combining these previous studies, it is suggested that the lack of significant anterior-posterior laxity in walking PCLD knees may be due to insufficient knee flexion angles.

During the swing phase of gait cycle, the involved knee exhibited smaller posterior tibial displacement compared to healthy controls. Additionally, the involved knee demonstrated significantly increased flexion angles at the initial stance phase, which should ideally be fully extended. Conversely, the flexion angle significantly decreased during the maximum swing phase flexion. This indicates a knee “protective state”, where patients constrain the range of movement. This observation aligns with the clinical walking characteristics of patients (Özbek et al., 2023; Nikkhoo et al., 2020). Cain and Schwab (1981) reported that, in football players with PCL injuries, the quadriceps muscle on the involved side contracted earlier in the gait cycle than in healthy conditions. The quadriceps insertion on the tibial tuberosity causes tibial anterior displacement when it contracts in a flexed knee position (Raghavan and Krishnakumar, 2023; Plastow et al., 2023). Tibone et al. (1988) utilized surface electromyography (sEMG) to assess muscle activation in PCLD patients and found earlier activation of the semimembranosus, gastrocnemius, and soleus muscles. Jonsson and colleagues also suggested that muscle activity can compensate for the functional absence of the posterior cruciate ligament (Jonsson and Kärrholm, 1999). Consequently, we propose that earlier muscle activation enhances dynamic knee stability, compensating for the instability caused by PCL deficiency.

The absence of instability during walking in PCL-deficient patients can be attributed to the low-load and low-flexion nature of walking, where knee flexion angles rarely exceed 60°. Studies (Castle et al., 1992; von Eisenhart-Rothe et al., 2012; Jonsson and Kärrholm, 1999) have shown that significant posterior tibial displacement typically occurs at higher flexion angles, such as during squatting or stair descent. Additionally, compensatory mechanisms, including increased quadriceps and hamstring activation and reduced posterior tibial displacement, contribute to maintaining dynamic stability during walking. Previous studies have shown that PCLD patients rely on synergistic activation of the quadriceps and hamstrings to suppress abnormal posterior tibial displacement during walking (Cain and Schwab, 1981; Raghavan and Krishnakumar, 2023; Plastow et al., 2023). This is achieved through the anterior shear forces generated by the quadriceps and the posterior stabilizing forces of the hamstrings, effectively maintaining knee stability. Moreover, early activation of the gastrocnemius and soleus muscles further enhances knee stability under dynamic conditions, reflecting an adjustment in neuromuscular control patterns (Jonsson and Kärrholm, 1999; Tibone et al., 1988).

However, the effectiveness of these compensatory mechanisms appears to be limited. While they maintain stability during low-load activities such as walking, the increased demands of high-load or high-flexion activities, such as stair descent, jumping, or rapid directional changes, may exceed the compensatory capacity, leading to potential instability. Therefore, clinical rehabilitation strategies should be optimized. Current conservative treatments for PCL injuries typically focus on strengthening key muscle groups, such as the quadriceps and hamstrings, gait training, and restricting high-demand activities (Lu et al., 2021; Crowell et al., 2017). However, these approaches often lack customization for the specific movement patterns and compensatory mechanisms associated with PCL deficiency. Our findings show that PCLD patients do not exhibit significant instability during walking but demonstrate distinct compensatory kinematic adjustments, such as increased knee flexion angles and reduced posterior tibial displacement. These compensations may be insufficient to maintain stability during higher-demand activities (Castle et al., 1992; von Eisenhart-Rothe et al., 2012; Jonsson and Kärrholm, 1999). Incorporating dynamic gait analysis to identify individualized compensatory mechanisms and designing targeted rehabilitation protocols—such as neuromuscular training, proprioceptive exercises, and gradually introducing high-demand activities like running or jumping—could enhance dynamic stability and better prepare patients for complex movement scenarios. Future research should focus on evaluating these interventions to refine conservative treatment strategies for PCLD patients.

Current diagnostic criteria for PCL injuries primarily rely on static anatomical assessments, such as the Posterior Drawer Test (Wollschläger et al., 2021) and MRI (Paletta and Warren, 1994), which confirm structural damage but fail to capture functional implications during dynamic activities. This study, consistent with previous findings (Fowler and Messieh, 1987; Shelbourne et al., 1999; Shelbourne and Muthukaruppan, 2005), reveals that while PCL-deficient patients exhibit structural deficits, they often maintain functional stability during walking. By examining 6DOF kinematics during level walking, we identified compensatory mechanisms, such as increased quadriceps activation and adaptive flexion patterns, that preserve dynamic stability. These results highlight the need for a multidimensional diagnostic approach that integrates dynamic biomechanical assessments, such as gait analysis, to evaluate knee kinematics, including anterior-posterior and rotational stability during daily activities. Differentiating between mechanical instability (Dhillon et al., 2011) and functional instability (Rivera-Brown et al., 2022) could provide deeper insights into these compensatory strategies and address the discrepancy between objective clinical examinations and patients’ subjective perceptions. Incorporating functional assessments into clinical protocols not only bridges the gap between structural findings and functional outcomes but also informs personalized treatment decisions, helping to identify patients at higher risk of instability during high-demand activities and guiding surgical versus conservative management strategies.

Several limitations should be noted in this study. First, the study did not incorporate additional specific and demanding motor tasks. Walking is a fundamental locomotor activity in daily life. Moreover, most patients reported an absence of joint instability during walking. Thus, we chose walking as the primary focus of our study. In future studies, we intend to develop more demanding motor tasks. Second, sEMG was not utilized in this study. sEMG measures surface electrical signals during muscle contraction, offering insights into muscle activation and performance during movement (Castle et al., 1992; Arthur et al., 2020). Currently, technical issues prevent us from synchronizing the 3D motion capture system with sEMG equipment, but once resolved, synchronized trials will be conducted. Lastly, analysis was limited to the knee joint. While knee instability is the primary concern in PCLD patients, our focus was mainly on the posterior displacement of the tibia relative to the femur. Previous studies, such as that by Gollehon et al. (1987), have explored adaptive changes in the hip and ankle joints in various movement dimensions, including flexion/extension, abduction/adduction, and internal/external rotation.

## 5 Conclusion

This study characterizes the movement patterns of both the involved and uninvolved legs during walking in patients with isolated PCL ruptures, and biomechanically confirms the absence of joint instability during walking in these patients. In comparison to healthy controls, the posterior displacement of the tibia in the involved leg was significantly reduced, along with a diminished range of knee flexion. Furthermore, clinical evaluations of PCLD should incorporate dynamic functional assessments, thereby providing a more comprehensive basis for treatment decisions.

## Data Availability

The raw data supporting the conclusion of this article will be made available by the authors, upon reasonable request.
